# The first molecular identification of benzimidazole resistance in *Haemonchus contortus* from goats in Thailand

**DOI:** 10.14202/vetworld.2021.764-768

**Published:** 2021-03-25

**Authors:** Opal Pitaksakulrat, Monticha Chaiyasaeng, Atchara Artchayasawat, Chatanun Eamudomkarn, Sorawat Thongsahuan, Thidarut Boonmars

**Affiliations:** 1 Department of Parasitology, Faculty of Medicine, Khon Kaen University, Khon Kaen 40002, Thailand; 2 Liver Fluke and Cholangiocarcinoma Research Institute, Faculty of Medicine, Khon Kaen University, Khon Kaen 40002, Thailand; 3 Faculty of Veterinary Science, Prince of Songkla University, Songkhla 90110, Thailand; 4 Neglected, Zoonosis and Vector-Borne Disease Research Group, Khon Kaen University, Khon Kaen 40002, Thailand

**Keywords:** benzimidazole resistance, *Haemonchus contortus*, single-nucleotide polymorphism in codon 200 β-tubulin isotype 1 gene

## Abstract

**Background and Aim:**

*Haemonchus contortus* is one of the major trichostrongyloid nematodes affecting small ruminant production worldwide, especially in tropical and subtropical regions. Adult *H. contortus* suck the blood from the host abomasum leading to anemia and often death in heavily infected animals. The mainstay of parasitic control is an anthelmintic drug, but long-term drug use may cause drug resistance. The aim of this study was to examine benzimidazole resistance in *H. contortus* of goats from different regions in Thailand by detecting the frequency of the F200Y polymorphism in the β-tubulin isotype 1 gene.

**Materials and Methods:**

A total of 121 *H. contortus* adults were obtained from 31 naturally infected out of 37 slaughtered goats from city abattoirs in five regions of Thailand. The frequency of the F200Y polymorphism in the β-tubulin isotype 1 gene was detected following the allele-specific polymerase chain reaction protocol.

**Results:**

The overall genotype frequencies in Thailand were homozygous resistant (RR: 24%), heterozygous (SR: 44.6%), and homozygous susceptible (SS: 31.4%). The allele frequencies were resistant allele (R: 46%) and susceptible allele (S: 54%). The R allele frequency and the RR genotype varied from 30% to 65% and 0% to 43.9%, respectively. The frequency of R alleles was significantly higher in the southern region (0.65) as compared to northern (0.30, p=0.001), western (0.38, p=0.04), and central regions (0.30, p=0.03). The RR genotype was also significantly higher in the southern region (43.9%) versus the northern (0 %, p=0.001), western (11.8%, p=0.012), and central regions (17.4%, p=0.001).

**Conclusion:**

This is the first study of the detection of single-nucleotide polymorphisms in codon 200 of the β-tubulin isotype 1 gene of *H. contortus* from goats in Thailand. These findings are essential and imply that an integrated approach is needed for issues such as drug treatment, farm management, prevention, and control strategies. This is of interest to farmers, veterinarians, and the department of livestock.

## Introduction

The livestock industry plays an important role in the agricultural economy. It has an average global growth rate of 40% of gross agricultural production in developing countries. They contribute to food security and nutrition, livelihoods, as well as national economic development. In Thailand, small ruminants such as goats are very popular because they are easy to manage and are in demand [1]. *Haemonchus contortus* is a trichostrongyloid nematode and an important parasitic disease that poses significant economic losses in small ruminants [2,3]. Small ruminants are infested with the worm when they graze with the infecting larvae (L3). After a host has ingested L3 larvae, the worm will burrow into the abomasum (true stomach) where it develops into an adult stage. Adult male and female worms live in the abomasum of the ruminants where they feed on blood. The adults feed on blood from host abomasa resulting in anemia and stunted growth, which can lead to edema until death in heavily infected animals [4]. Several studies have reported this parasite in Thailand [5-7]. Moreover, in terms of zoonotic parasite, many studies have also reported that *H. contortus* can infect humans [8-10] and this supports the One Health approach concern.

The control of hemonchosis is mostly based on anthelmintic treatment (ATH), including benzimidazole, but this may increase the emergence of anthelmintic resistance. Many countries such as Brazil, China, India, Pakistan, and the United States of America have reported anthelmintic resistance of this parasite to all major classes of ATH drugs [11-21]. Benzimidazole (BZ) resistance has been associated with three different mutations in the β-tubulin isotype 1 gene in *H. contortus* with single-nucleotide polymorphism (SNPs). These mutations include the replacement of a phenylalanine (Phe, TTC) by a tyrosine (Tyr, TAC) at positions 200 [22] and 167 [23] and rare SNPs that alters Ala (GCA) to Glu (GAA) at position 198 of β-tubulin isotype 1 gene [24].

Thus, the aim of this study was to examine benzimidazole resistance in *H. contortus* of goats from different regions in Thailand by detecting the frequency of the F200Y polymorphism in the β-tubulin isotype 1 gene. The data generated from this study are essential for understanding the status of BZ resistance and may integrate approaches for farm management and ATH program, thus reducing the economic losses from anthelmintic resistance in Thailand.

## Materials and Methods

### Ethical approval

No ethical approval required for this study because *H. contortus* samples were collected from slaughtered animals.

### Study period, area, and sampling

**Figure-1:**
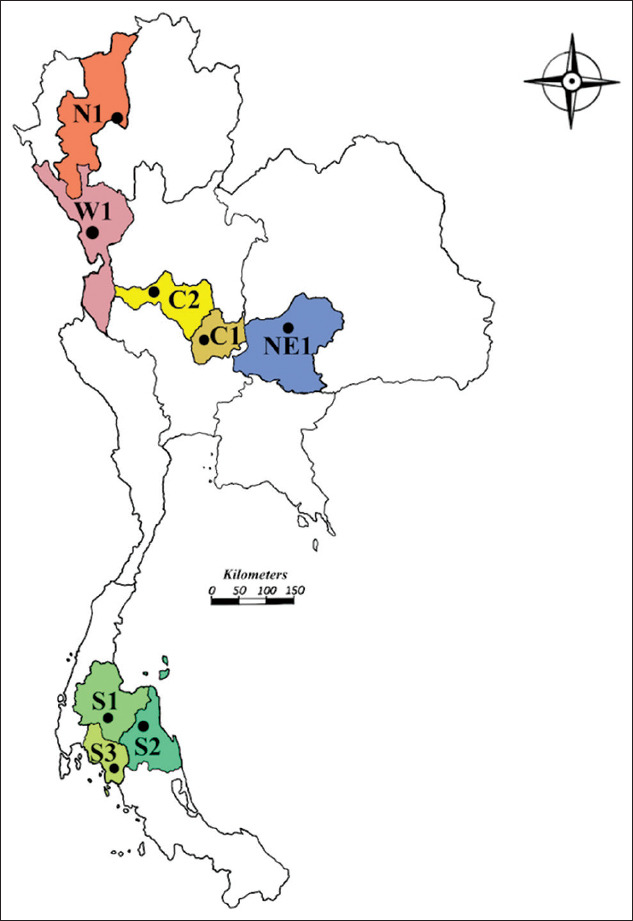
*Haemonchus contortus* adult worms were collected from goats in eight city abattoirs from five different regions of Thailand (Thailand map was modified from https://go.gistda.or.th).

This study was conducted from January 2019 to February 2020. *H. contortus* worms were recovered from 31 naturally infected out of 37 small ruminant abomasa from city abattoirs from eight provinces in five different regions of Thailand (the north, west, central, northeast, and south; Table-1 and Figure-1). Each abomasum was opened, and the contents were washed into a glass beaker. The worms were morphologically identified at the genus level *Haemonchus* spp. [25]. Male and female *Haemonchus* were identified at the species level under a microscope [2,26]. All worms were thoroughly washed in 0.85% physiological saline and then stored at −20°C until used for molecular analysis.

**Table-1:** *Haemonchus contortus* adult worms were collected from goats in eight city abattoirs from five different regions of Thailand.

Regions	Province/code	No. of abomasum	Geographical localities
North	Chiang Mai/N1	3	18°46’40.7”N 98°59’52.8”E
West	Tak/W1	5	16°45’03.8”N 98°30’36.5”E
Northeast	Nakhon Ratchasima/NE1	8	14°59’10.7”N 102°06’19.1”E
Central	Lopburi/C1	6	14°47’43.5”N 100°40’14.2”E
	Nakhon Sawan/C2	1	15°43’06.1”N 100°06’52.8”E
South	Surat Thani/S1	1	8°38’28.4”N 99°20’02.5”E
	Nakhon Si Thammarat/S2	3	8°38’39.6”N 99°56’45.6”E
	Krabi/S3	4	7°38’10.5”N 99°06’40.8”E
Total	8	31	

### Isolation of genomic DNA

Male and female worms from each abomasum were pooled for each population of *H. contortus* from eight provinces in the five regions. The male and female adults of *H. contortus* were randomly selected from the pooled sample of each of the eight populations for detecting the BZ resistance analysis. The genomic DNA was extracted from 121 individual male and female worms from eight populations using the DNeasy blood and tissue kit (QIAGEN Ltd., Crawley, West Sussex, UK). The DNA was eluted in a total volume of 25 μL. The concentration and quality of the extracted DNA were then measured in a Nanodrop spectrophotometer.

### Allele-specific polymerase chain reaction (AS-PCR) amplification

To examine the frequency of the F200Y polymorphism in the β-tubulin isotype 1 gene, the resistant (R) and susceptible (S) alleles were explored following the AS-PCR protocol [27]. The AS-PCR was amplified in single reaction using the four primers as follows: Non-AS PH1 (5′-GGA ACG ATG GGA CTC CTT TCG-3′), susceptible AS PH4 (5′-ATA CAG AGC TTC GTT GTC AAT ACA AG-3′), resistant AS PH3 (5′-CTG GTA GAG AAC ACC GAT GAA ACA-3′), and non-AS Pn2 (5′-GAT CAG CAT TCA GCT GTC CA-3′). The PCR mixture has a total volume of 25 μL using illustra™ pureTaq Ready-To-Go PCR Beads (GE Healthcare, UK), 10 pmol of each primer, and 2.0 μL of DNA template (50-100 ng/μL). The following PCR conditions were used: Initial denaturation at 94°C for 15 min, 40 cycles of denaturation at 95°C for 1 min, annealing at 60°C for 1 min, and extension at 72°C for 1 min; the final elongation step was at 72°C for 10 min. After AS-PCR amplification, allele-specific analysis used electrophoresis in 2% agarose gel stained with ethidium bromide. The susceptible, resistant, and non-specific bands were observed at approximately 550, 250, and 650 bp, respectively. The genotypes of adults were homozygous resistant (RR), homozygous susceptible (SS), and heterozygous (SR).

### Statistical analysis

A Chi-square test compared the allele and genotype frequencies of adult *H. contortus* from five different regions [28].

## Results

**Figure-2:**
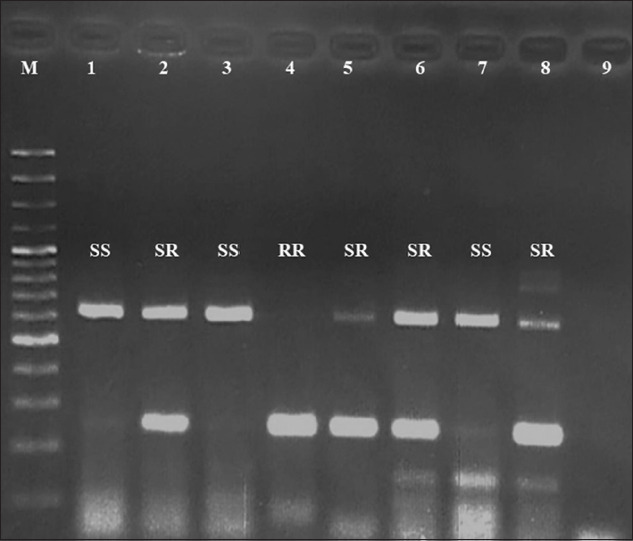
Polymerase chain reaction product of β-tubulin gene from *Haemonchus contortus*, lane M is DNA ladder (100 bp), lane 1-8 is PCR product of β-tubulin gene, lane 9 is negative control. The genotype; homozygous resistant (RR), homozygous susceptible (SS), and heterozygous (SR).

There were 121 male and female *H. contortus* adults from eight populations in five regions – these were genotyped to detect SNPs in codon 200 of the β-tubulin isotype 1 gene. The allele and genotype frequencies are given in Table-2 and Figure-2. Three genotypes (RR, SR, and SS) were detected. The overall genotype frequencies of *H. contortus* adults in Thailand were homozygous resistant (RR: 24%), heterozygous (SR: 44.6%), and homozygous susceptible (SS: 31.4%). The allele frequencies were resistant allele (R: 46%) and susceptible allele (S: 54%).

**Table-2:** Genotype and allele frequencies associated with BZ in *Haemonchus contortus* adult from goats in eight provinces of five regions, Thailand.

Regions	Province	Number of worm in each pool	Genotype frequency (%)			Allele frequency	
	
			RR	SR	SS	Resistant (R)	Susceptible (S)
North	Chiang Mai	20	0	12 (60.0)	8 (40.0)	0.30*	0.70*
West	Tak	17	2 (11.8)*	9 (52.9)	6 (35.3)*	0.38*	0.62*
Northeast	Nakhon Ratchasima	20	5 (25.0)	10 (50.0)	5 (25.0)	0.50	0.50
Central	Lopburi	20	3 (15.0)*	6 (30.0)	11 (55.0)*	0.30	0.70
	Nakhon Sawan	3	1 (33.3)	0	2 (66.7)	0.30	0.70
	Total	23	4 (17.4)*	6 (26.1)	13 (56.5)*	0.30*	0.70*
South	Surat Thani	9	8 (88.9)	1 (11.1)	0	0.94	0.06
	Nakhon Si Thammarat	15	5 (33.3)	7 (46.7)	3 (20.0)	0.57	0.43
	Krabi	17	5 (29.4)	9 (53.0)	3 (17.6)	0.56	0.44
	Total	41	18 (43.9)*	17 (41.5)	6 (14.6)*	0.65*	0.35*
Total	121	29 (24.0)	54 (44.6)	38 (31.4)	0.46	0.54

* Indicates significance at P*<*0.05

The homozygous resistant (RR) had high frequencies (25-43.9%) in the northeast and southern regions. They were low (0-17.4%) in three others (north, west, and central regions). The genotype that was heterozygous (SR) and homozygous susceptible (SS) was detected in all regions ranging from 26.1% to 52.9% and 14.6% to 56.5%, respectively. The resistance genotype (RR) was also significantly higher in the southern region (43.9%) versus the northern (0%, p=0.001), western (11.8%, p=0.012), and central regions (17.4%, p=0.001).

The resistance alleles frequency associated with BZ in *H. contortus* from eight provinces in five regions varied from 30% to 65% and the susceptible alleles frequency ranged from 35% to 70%. The highest resistant alleles (R) frequency (65%) was in the south. The frequency of the R alleles was significantly higher in the southern region (0.65) versus the northern (0.30, p=0.001), western (0.38, p=0.04), and central regions (0.3, p=0.03).

## Discussion

This is the first evidence of BZ resistance from *H. contortus* in Thailand by screening the frequency of the F200Y polymorphism in the β-tubulin isotype 1 gene. The objective of this study was to examine SNPs in codon 200 of *H. contortus* adults from different regions in Thailand using the AS-PCR technique. This technique used four primers in a single reaction to save time and reagent costs. Moreover, this technique is easy and rapid to determine the BZ resistance in *H. contortus*. We detected a high frequency of resistant homozygous RR in the southern region (65%) similar to the previous studies in China (31%), Egypt (69.44%), Northwest India (74%), North India (53-85%), Hungary (87.2%), Brazil (66.7-93.97%), India (98%), and East Amazon (100%) [14,29-35]. The low frequencies of resistance allele (0-15%) in *H. contortus* in three others (the northern, western, and the central regions) are similar to a study in Brazil (5%) [36]. This may be because of lower use of ATH drug by farmers and low pasture contamination with the other ruminants [1,33].

In Thailand, the treatment of hemonchosis mostly uses two groups of broad-spectrum anthelmintic drugs, including benzimidazole and avermectins. Thai veterinarians and farmers often use BZ such as albendazole, for an extended period of time. The long-term administration of the same drug (albendazole) could change the relative allele frequency of β-tubulin isotype 1 genes associated with BZ resistance in this parasite [1,31,33].

In the south, the frequency of resistance alleles (R) and genotypes (RR) was higher than other regions, perhaps because of frequent dosing, under dosing of BZ drug, and high pasture contamination with the other ruminants such as cattle in the same area. Moreover, small ruminant agriculture, including goats is mainly located in the south because this animal is popular and important for Muslim people (goats are associated with the Muslim religion and culture) [1]. Goats have been raised for generations so the worm may be in contact with ATH drug for a long time leading to resistance [31,33]. These results confirm the rapid emergence and selection of allele resistance in South Thailand.

## Conclusion

This is the first molecular identification of BZ resistance (SNPs in codon 200 of the β-tubulin isotype 1 gene in *H*. *contortus*) from goats in Thailand. Our results showed that three genotypes (RR, SR, and SS) were detected in five regions. The high resistance allele frequency and the resistance genotype in codon 200 of the β-tubulin isotype 1 gene of *H. contortus* from goats were observed in South Thailand. These data are essential for understanding BZ resistance in Thailand and may integrate approaches for farmers, veterinarians, and the government. Farm management could include ATH drug treatment or grassland rotation plans to reduce the economic losses from anthelmintic resistance.

## Authors’ Contributions

OP, MC, AA, ST, and TB: Data curation, investigation, methodology, and formal analysis. OP and TB: Conceptualization and project administration. TB: Supervision and visualization. OP, AA, CE, and TB: Drafted and revised the manuscript. All authors read and approved the final manuscript.
